# 

**Published:** 1885-04

**Authors:** 


### NEW BOOKS AND PAMPHLETS.

A TEXT-BOOK OF HYGIENE, a comprehensive treatise on the principles and practice of preventive medicine from an American standpoint, by George H. Rohie, M. D., of Baltimore. Thomas & Evans, publishers. Price, $3.00.

Much has been written upon the subject of public hygiene of late, the theme possessing renewed interest by reason of the prospective visitation of cholera to our shores this summer. In this book of Dr. Rohie's is embodied all that is worth knowing of sanitary and hygienic methods that are likely to be available in the present emergency. It is a book of real merit, written by one who has had much practical experience in promulgating the theories of which he writes.

THE MICROSCOPE, an illustrated monthly journal edited and published by Charles H. Stowell, M. D., F. R. M. S., and Louisa Reed Stowell, M. S., F. R. M. S., Ann Arbor, Mich. $1.00 a year.

The only journal devoted solely to microscopy, in this country, that can lay any claim to genuine merit.

REPORT OF COMMITTEE ON SCHOOL HYGIENE IN TENNESSEE. By Daniel F. Wright, M. D., of Clarksville, Tenn. With a report of a sanitary inspection of the public schools. Dr. Wright proves himself to be thoroughly conversant with school sanitation and his monograph is therefore worthy of careful consultation.

